# Buffalo State Asylum for the Insane—The Opening

**Published:** 1880-10

**Authors:** 


### BUFFALO STATE ASYLUM FOR THE INSANE.—THE OPENING.

It is confidently expected that this important institution will soon be ready for occupancy, and will open for the reception of patients sometime during the present month. The Superintendent, Dr. Judson B. Andrews, has been in the city making the necessary preparations for inaugurating the work, with such success, that the Board of Managers anticipate that the labors, incident to the construction of these extensive buildings, will be so far completed, as to enable them to witness the realization of their hopes at an early day.

We have the utmost confidence that in the selection of subordinates for the various positions required in such institutions, the same wise judgment will be exercised as inspired the appointment of the able Superintendent.

The profession of the state, and especially of Buffalo, have a just pride in this institution, and with its modern appointments and able medical corps, feel assured that it will at once command the leading position in the treatment of this class of diseases.

